# Structure Determination of Europium Complexes in Solution Using Crystal-Field Splitting of the Narrow *f*–*f* Emission Lines

**DOI:** 10.1021/acs.jpclett.1c01885

**Published:** 2021-07-19

**Authors:** Yoshinori Okayasu, Junpei Yuasa

**Affiliations:** Department of Applied Chemistry, Tokyo University of Science, 1-3 Kagurazaka, Shinjuku-ku, Tokyo 162-8601, Japan

## Abstract

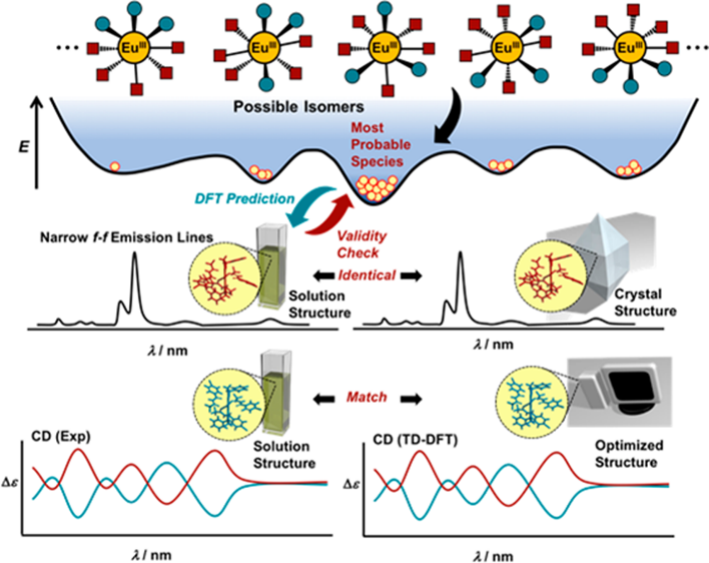

Nine nona-coordinated Eu(III) complexes (**1**–**9**) studied here have three unsymmetric β-diketonate ligands and one chiral Ph-Pybox ligand, which can produce eight possible coordination isomers, depending on the position of the three unsymmetric β-diketonate ligands. Substituents on the β-diketonate ligands cause a rational structural rearrangement upon crystallization. Substituents with higher polarity, including −CN, −F, −Cl, −Br, −OMe, and −OEt, employ intercomplex hydrogen bonding to generate an association complex through structural rearrangement upon crystallization. Substituents with lower polarity, including −CF_3_, −SMe, and −Me, cause the most energetically favorable isomer to crystallize directly from solution. These two crystal structures exhibit well-resolved *f–f* emission lines with characteristic Stark splitting structures. This work revealed that the configuration of the Eu(III) complexes in solution can be determined by systematic comparison of their Stark splitting structures to those obtained from the solid phase using density functional theory (DFT)-based predictions combined with circular dichroism data.

The determination of the configuration of coordination complexes has a major impact on various fields of chemistry. In this context, single-crystal X-ray diffraction analysis is the most common method for addressing structure determination in the solid state. However, structure determination of kinetically labile species using this technique is always challenging because such species easily undergo structural rearrangements upon crystallization, and the configurations of the solid-state crystal structures are often different from those that exist in solution.^1−5^ In particular, lanthanide ions are extremely labile and have versatile coordination numbers (*n* ≥ 8),^3−7^ giving rise to dynamic ensembles of coordination isomers that coexist in solution.^8−10^ Furthermore, most lanthanide ions are paramagnetic, making NMR-based structure determination of their complexes challenging.^3−5,11^ Such analytical complexity presents a bottleneck for developing specific lanthanide frameworks specifically in solution, which should exhibit fascinating physical properties such as molecular magnetism,^12,13^ the ability to function as emission sensors^14−21^ or photoswitch devices,^22−25^ and the generation of circularly polarized luminescence.^26−46^

Herein, we propose a protocol for the determination of the configurations of europium(III) [Eu(III)] complexes in solution using crystal-field splitting of the narrow *f*–*f* emission lines with appropriate use of density functional theory (DFT)-based structure prediction and feedback (see details in Scheme 1). Lanthanide complexes often exhibit well-resolved emission lines arising from transitions between the inner-shell *f*-orbitals; their *f*–*f* emission lines split into several Stark levels because of the crystal field.^47^ Such unique photophysical properties of lanthanide ions enable the complexes to exhibit emission lines that are characteristic of individual differences in coordination structure.^48−50^ In particular, ^5^D_0_ → ^7^F_2_ transition of Eu(III) is universally called “hypersensitive transition” and is widely used to obtain insight into structures of Eu(III) complexes. To verify the proposed approach (Scheme 1), we synthesized a total of nine nona-coordinated Eu(III) complexes^8,9,41−46,51,52^ (**1**–**9**) as representative complexes, the solid-state configurations of which were successfully determined by X-ray crystallography. The present series of complexes contains three unsymmetric β-diketonate ligands (O^O′) and one chiral Ph-Pybox ligand (N^N^N*).^8,9,41−46,53^ The resulting (N^N^N*)(O^O′)_3_-type nona-coordinated Eu(III) complex can produce eight possible coordination isomers (isomers **A**–**H**) depending on the spatial relationships of the three unsymmetric β-diketonate ligands (Figure 1). Here, we attached electron-withdrawing or -donating substituents to the unsymmetric β-diketonate ligands to offer a rational structural rearrangement upon crystallization, in which higher-polarity substituents (−CN, −F, −Cl, −Br, −OMe, and −OEt) provide scaffolds employing intercomplex hydrogen bonding to generate an association complex through the structural rearrangement of isomer **G** to **H** upon crystallization. The lower-polarity substituents (−CF_3_, −SMe, and −Me) have no such effect on crystallization; therefore, the most stable isomer, isomer **G**, crystallized directly from solution. The different, characteristic Stark splitting structures in the well-resolved *f–f* emission lines of the two types of crystal structures obtained for isomers **G** and **H** will provide a valuable insight into the elucidation of the structure of the Eu(III) complexes in solution.

**Scheme 1:**
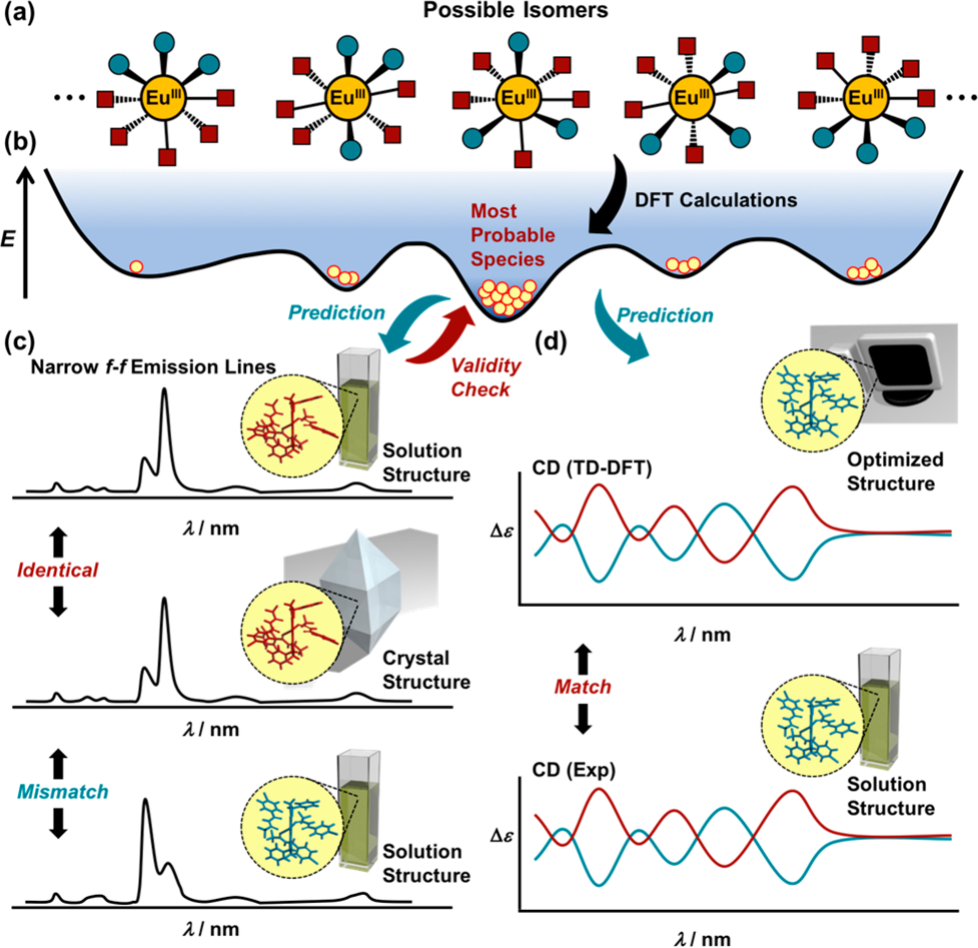
Proposed Protocol for Determination of Configuration of Eu(III) Complexes in Solution Phase (a) All of the possible coordination isomers of the Eu(III) complexes. (b) Preparation of the energy potential surface of the possible isomers by using density functional theory (DFT) calculations for the DFT-based structure prediction and feedback. (c) Comparison of the characteristic Stark splitting structures obtained from the solid and solution phases. If the two “fingerprint-like” emission profiles are identical, the solution structure should be the same as that determined by X-ray crystallography. Conversely, if the two profiles are mismatched, structural rearrangement presumably occurred upon crystallization. (d) Comparison of the experimentally obtained circular dichroism (CD) spectrum and the theoretical CD spectrum of the probable isomers obtained with time-dependent (TD) DFT.

**Figure 1:**
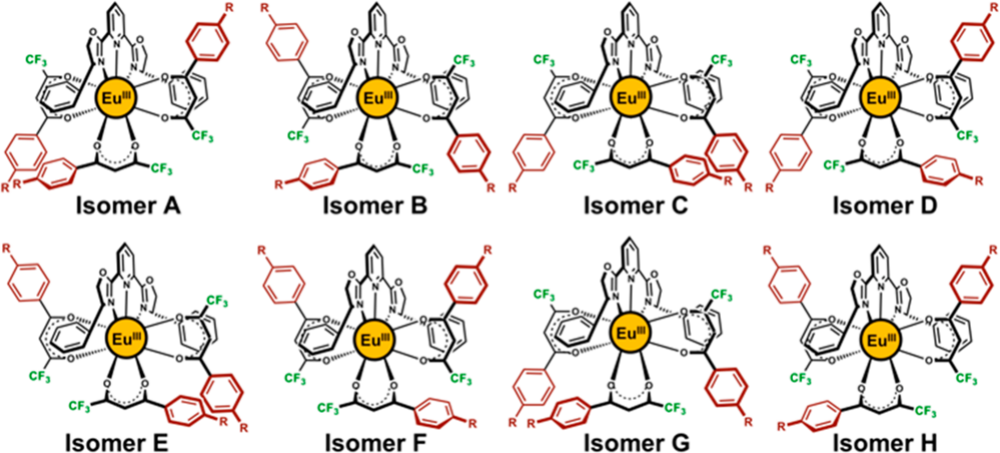
Possible coordination isomers of (N^N^N*)(O^O′)_3_-type nona-coordinated Eu(III) complexes [R = −CN (**1**), −CF_3_ (**2**), −F (**3**), −Cl (**4**), −Br (**5**), −SMe (**6**), −Me (**7**), −OMe (**8**), and −OEt (**9**)].

The (N^N^N*)(O^O′)_3_-type nona-coordinated Eu(III) complexes were synthesized by the reaction of the corresponding tris-β-diketonate Eu(III) complexes and a chiral Ph-Pybox ligand (*R*-form) in a 1:1 ratio in methanol (see details in the Supporting Information).^44^ In accordance with our proposed protocol (Scheme 1, *vide supra*), we performed DFT calculations to predict the potential energy surfaces for the eight possible isomers of the (N^N^N*)(O^O′)_3_-type nona-coordinated Eu(III) complexes (Figure 1, isomers **A**–**H**). To minimize the cost of calculations, we chose the nona-coordinated complexes having −CF_3_ (**2**) and −OMe (**8**) as typical examples of electron-withdrawing and -donating effects on the complexes, respectively. The nona-coordinated Eu(III) complexes optimized with DFT [DFT/CAM-B3LYP/def2SVP (ligands)/def2TZVPP (La)] can reproduce well the corresponding X-ray crystal structures, underlining the validity of the above DFT results (insets of Figure 2, *vide infra*).^46^ The DFT-estimated potential energy surface is visualized in Figure 2 (top), which demonstrates that isomer **G** is the energetically most stable isomer for both complexes **2** and **8***in vacuo*. Although a subtle energetic preference for isomer **G** over the other isomers (0.6–7.2 kcal mol^–1^) could be difficult to rationalize, isomer **G** looks the most symmetric among the eight possible isomers (Figures 1 and 2). Furthermore, solvation effects were considered for each isomer using the polarizable continuum model (IEFPCM: acetonitrile), which can produce a reasonable solvation energy ranging from 16.6 to 25.0 kcal mol^–1^ (Figure 2a,b, from top to bottom). Consequently, differences in energy of **A** through **H** isomers are less than 5.4 kcal mol^−1^ in solution, while isomer **G** remains energetically the most stable (Figure 2a,b, bottom). Thus, the DFT calculations predict that isomer **G** is the most probable species in both the solid and solution phases, irrespective of the electron-withdrawing or -donating nature of the substituents on the β-diketonate ligands.

**Figure 2:**
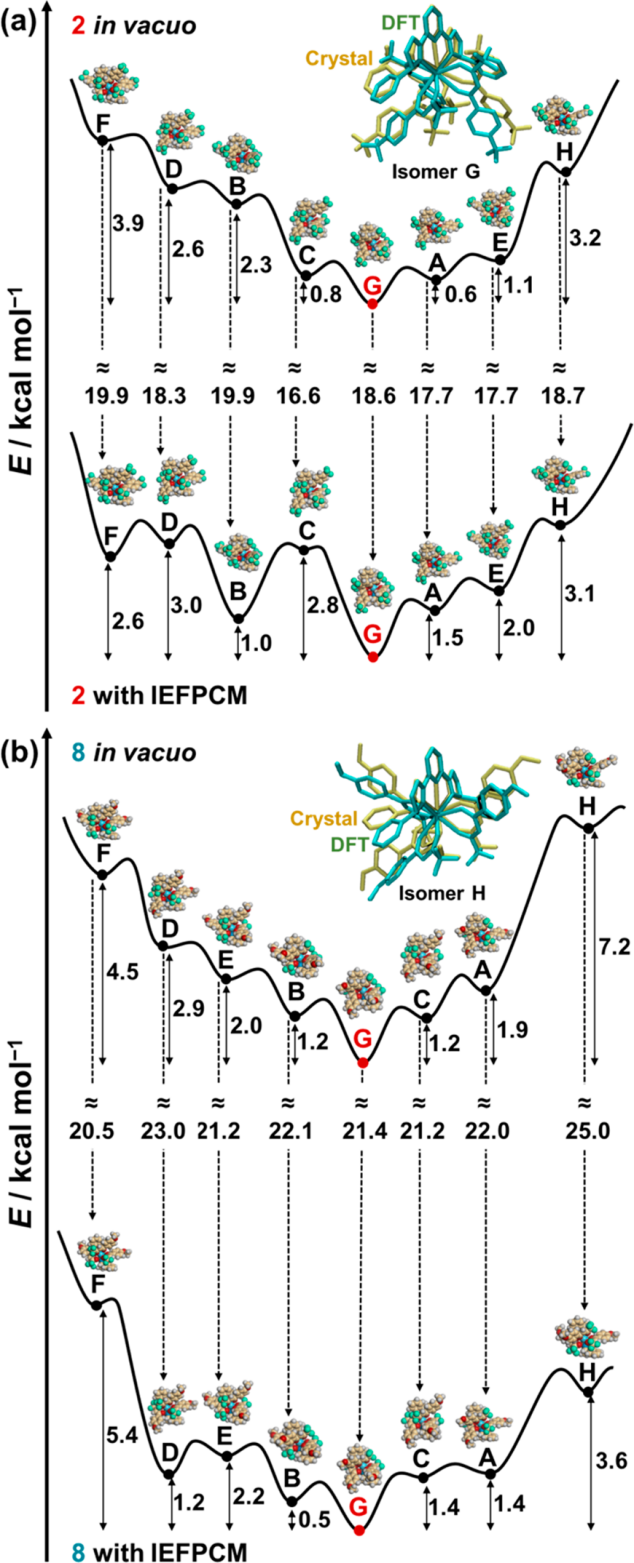
Potential energy surfaces for the isomers **A**–**H** of (a) **2** and (b) **8** optimized with DFT/CAM-B3LYP/def2SVP (ligands)/def2TZVPP (La) *in vacuo* and IEFPCM: acetonitrile, replacing Eu atoms with La atoms to reduce the calculational complexity. Potential energy points are connected with smooth lines for clarity. Insets show the overlapped image between the crystal structures (yellow) and the DFT-optimized structures (green) of (a) **2** and (b) **8**, omitting hydrogen atoms for clarity.

In light of the above DFT resuluts, we then performed X-ray structure analyses, by which the solid-state configurations of all nine nona-coordinated Eu(III) complexes (Eu-**1**–**9**) were successfully determined (Figure 3 and Tables S1 and S2). Suitable crystals can be grown from methanol or acetonitrile solutions of the Eu(III) complexes through slow evaporation. The X-ray structure analyses of the Eu(III) complexes revealed that the complexes containing the lower-polarity substituents— −CF_3_, −SMe, and −Me (Eu-**2**, **6**, and **7**, respectively)—crystallized as isomer **G** (Figure 3), the most stable species predicted by the above DFT studies (*vide supra*, Figure 2). The Eu(III) complexes **1**, **3**, **4**, **5**, **8**, and **9** with higher-polarity substituents (−CN, −F, −Cl, −Br, −OMe, and −OEt, respectively) crystallized as isomer **H**, in which extended intercomplex hydrogen bonding was found between the polar substituents attached to the β-diketonate ligands and the aliphatic hydrogen atoms of the Ph-Pybox ligands (Figures 3 and S1). In addition to the intercomplex hydrogen bonding, an intercomplex π–π stacking interaction was found between the benzene rings of the β-diketonate ligands, which makes the association complex robust (Figure 3).^54,55^ Although isomer **H** is suggested to be 3.1–3.6 kcal mol^–1^ higher in energy than the most stable isomer **G** by the above DFT studies (Figure 2, *vide supra*), isomer **H** appears to be the more suitable configuration for the intercomplex association (Figures 3 and S1). Thus, the solid-state structure of isomer **H** found for **1**, **3**, **4**, **5**, **8**, and **9** is presumably the result of compensatory energy gain due to the intercomplex interactions formed specifically in the solid state, inducing the structural rearrangement of the Eu(III) complexes during the crystallization process. To estimate the energy gain arising from the intercomplex interations, a dimeric complex of **8** with the geometry of isomer **H** (**8**_**H**_···**8**_**H**_) was optimized with DFT [DFT/CAM-B3LYP/def2SVP (ligands)/def2TZVPP (La)], and its potential energy was compared with that of the monomer complex (**8**_**H**_). The DFT results suggest a 5.0 kcal mol^–1^ energy gain *in vacuo* per intercomplex assocation (Figure S2), which could overcome the energetic preference of the most stable isomer **G** over isomer **H**.

**Figure 3:**
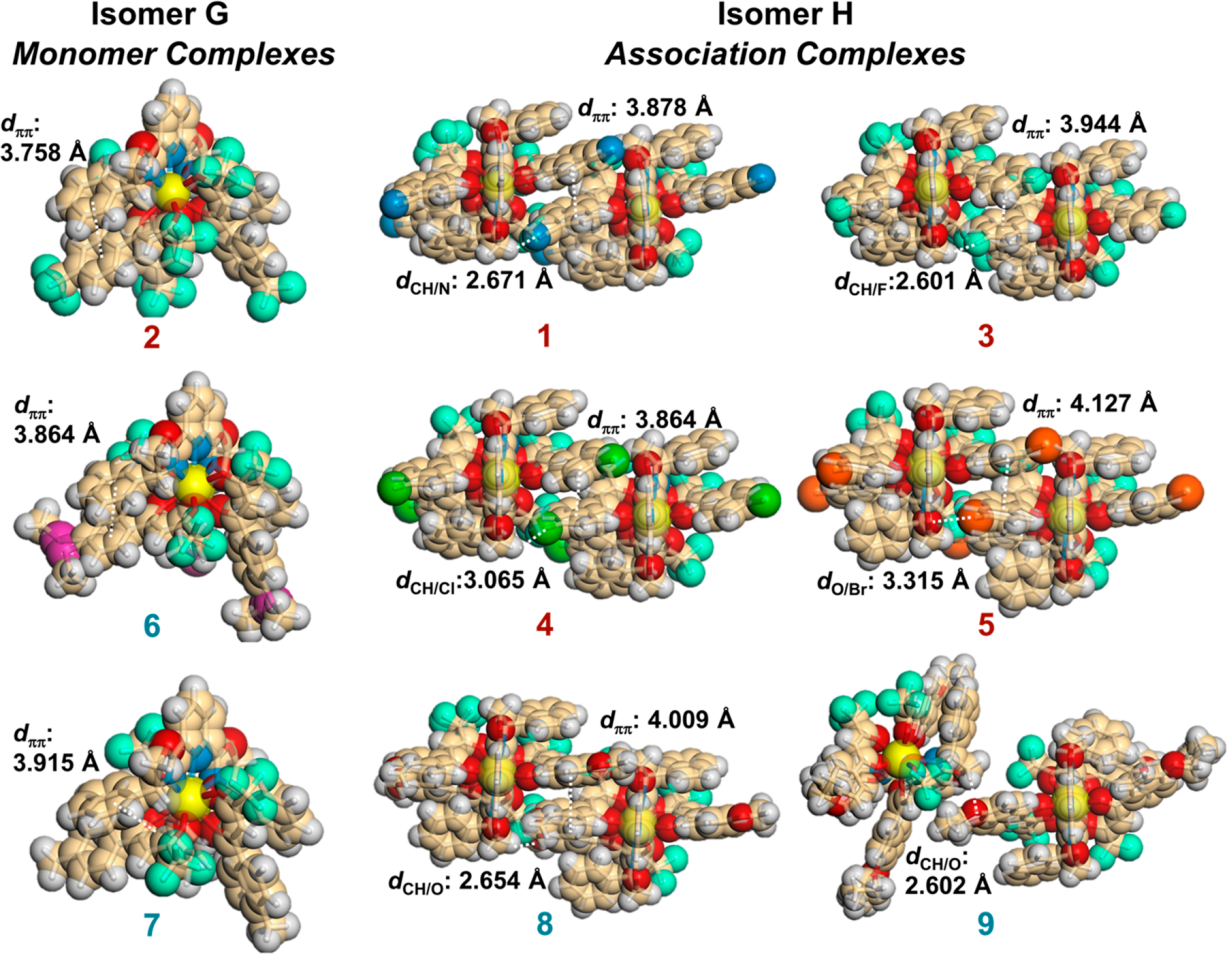
X-ray crystal structures of **1**–**9**. Selected intra- and intercomplex interactions are visualized (CCDC 2086828–2086836).

With these results, according to the proposed protocol (Scheme 1, *vide supra*), we determined the configuration of Eu(III) complexes in solutions of toluene or acetonitrile using crystal-field splitting of the narrow *f*–*f* emission lines. Figure 4 summarizes the comparison of solid- and solution-state emission spectra of complexes **1**–**9** in the region of the *f*–*f* transition of Eu(III) (^5^D_0_ → ^7^F_*n*_, *n* = 0–4). Both types of crystal structures (isomers **G** and **H**) show well-resolved *f*–*f* emission lines; however, their Stark splitting structures differ, particularly for the ^5^D_0_ → ^7^F_2_ transition (panels a and c of Figure 4, respectively). Isomer **G** shows two Stark splitting levels of the ^5^D_0_ → ^7^F_2_ transition band, in which weak and strong emission lines appear at λ_em_ = 613 and 620 nm, respectively (Figures 4a and S3). Isomer **H** has three Stark splitting lines at λ_em_ = 612, 615, and 621 nm, with similar emission intensities (Figures 4c and S3). Hence, although the isomers **G** and **H** have the same (N^N^N*)(O^O′)_3_-type coordination formula, they exhibit individual differences in the Stark splitting structures of the narrow *f*–*f* emission specific to the different orientations of the unsymmetrical β-diketonate ligands (for shape measure analyses of their coordination geometry, see Table S3 and S4).^54,55^ When the resulting solid-state emission profiles corresponding to isomers **G** and **H** were compared with those obtained in the nonpolar solvent toluene, all Eu(III) complexes (**1**–**9**) were found to exhibit *f*–*f* emission lines with Stark splitting structures identical to those of isomer **G** in the solid state (panel b vs a of Figure 4). Thus, we can successfully identify the predominant species of **1**–**9** in toluene as isomer **G**, as predicted by the above DFT calculations (*in vacuo*). By comparison, in the polar solvent acetonitrile, the Stark splitting structures of **1**–**9** (except **2**) were markedly different from those of isomers **G** and **H** in the solid state (panel d vs panels a and c of Figure 4), showing an enhancement of the Stark splitting line at λ_em_ = 613 nm with increasing electron-donating ability of the substituents on the β-diketonate ligands (Figure 4d). Thus, the observed marked difference in the Stark splitting structures enables us to rationalize that **1**–**9** (except **2**) contain competing isomers other than isomers **G** and **H**. The Stark splitting structures of **8** observed in the polar solvent became close to those of isomer **G** in the solid state with a decrease in temperature (Figure S4), indicating that the competing isomers coexist in equilibrium with isomer **G**. The solid- and solution-state structures of **1**–**9** are summarized in Scheme 2. In a nonpolar solvent, all complexes exist as the most stable isomer **G**, irrespective of the electron-withdrawing or -donating nature of the substituents (Scheme 2a). In a polar solvent, **1**–**9** (except **2**) give rise to competing isomers in equilibrium with isomer **G**; the relative ratio of the competing isomers to isomer **G** increases with increasing electron-donating ability of the substituent (Scheme 2c,d). In contrast, **2**, with a strongly electron-withdrawing group (R = −CF_3_), preserved the structure of isomer **G** even in a polar solvent (Scheme 2b, *vide supra*). In the solid state, among the Eu(III) complexes studied here, **1**, **3**, **4**, **5**, **8**, and **9**, with substituents capable of forming intercomplex hydrogen bonding (R = −CN, −F, −Cl, −Br, −OMe, and −OEt, respectively), underwent structural rearrangement to isomer **H** upon crystallization (Scheme 2f). The complexes without hydrogen-bonding electron-donating groups, **2**, **6**, and **7** (R = −CF_3_, −SMe, and −Me, respectively), crystallized from solution as isomer **G** (Scheme 2e).

**Figure 4:**
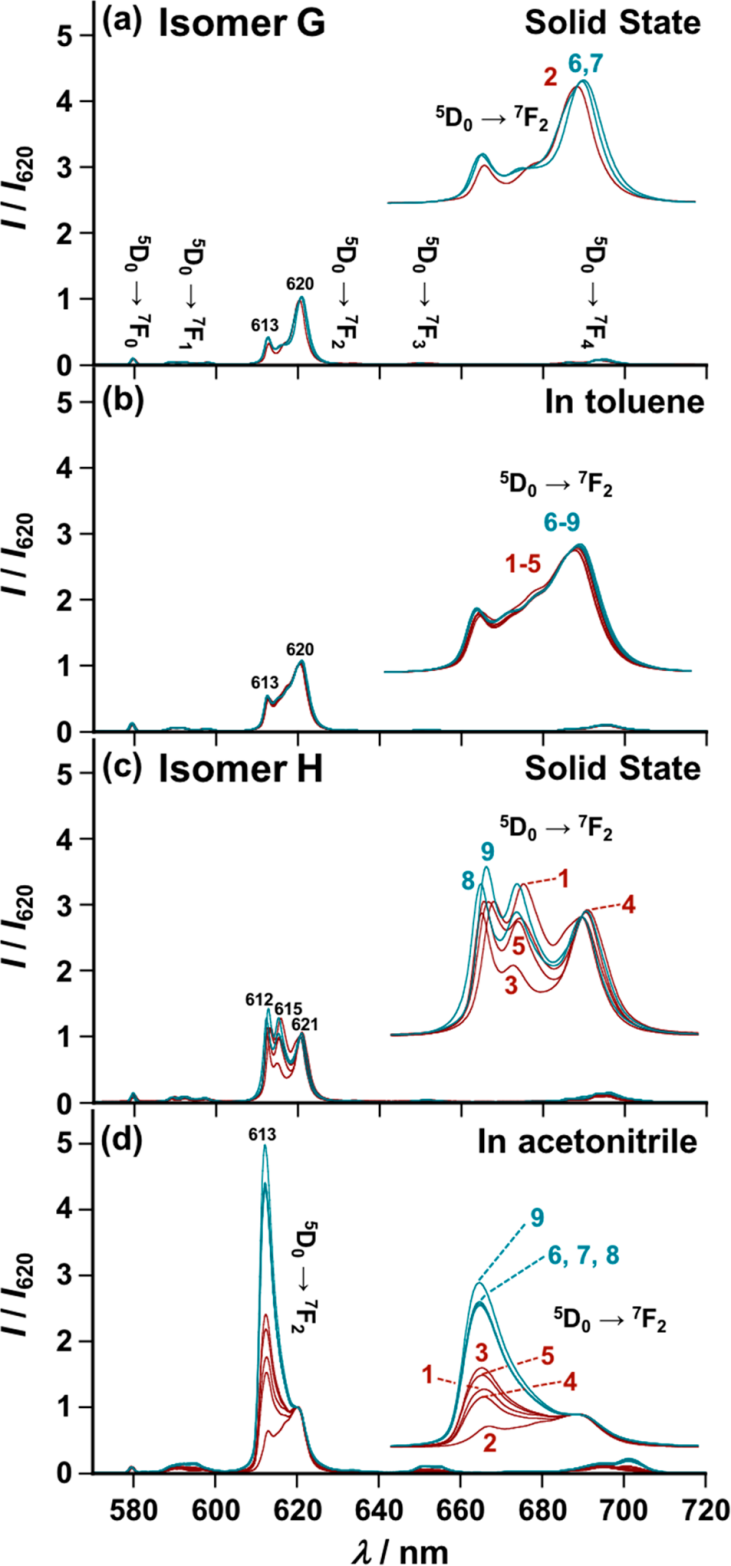
(a and c) Solid-state emission spectra (KBr) of (a) **2**, **6**, and **7** and (c) **1**, **3**, **4**, **5**, **8**, and **9**. (b and d) Emission spectra of **1**–**9** (concentrations: 1.0 × 10^–5^ M) in (b) toluene and (d) acetonitrile. Insets: (a–d) Emission bands at ^5^D_0_ → ^7^F_2_ are expanded.

**Scheme 2:**
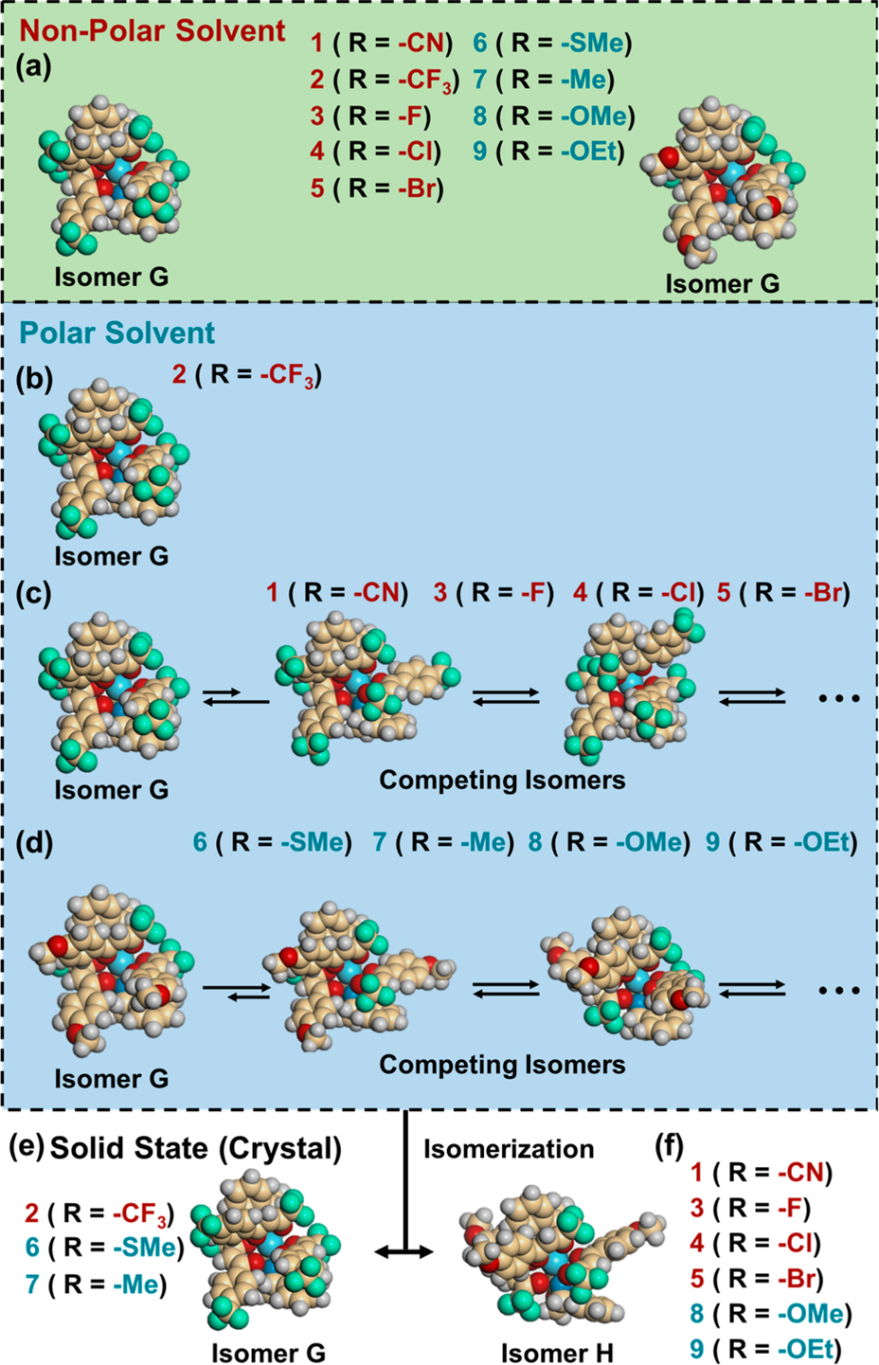
Summary of Solid- and Solution-State Structures of **1**–**9**

Next, we performed a time-dependent (TD) DFT-based structure elucidation for species in solution (*vide infra*). Because of the intracomplex ligand-to-ligand interaction (π–π stacking) between the β-diketonate ligand and the Ph group of the chiral Ph-Pybox (*R*-form) ligand (Figure 3), **2** (R = −CF_3_) gives rise to a chiral configuration for the three β-diketonate ligands around the Eu(III) metal center (Figure 5b). Consequently, **2** exhibited the characteristic biphasic CD spectra (Figure 5a) arising from excitonic coupling between the chromophoric ligands (Figure S5). Because excitonic coupling is sensitive to the distance and orientation of the chromophoric ligands located in the coordination sphere, the characteristic biphasic CD patterns influence the structure elucidation of the Eu(III) complexes in solution, not only in this case but also for other chiral complexes containing chromophoric ligands.^56−58^ During this study, we realized that the TD-DFT calculations obtained with DFT/CAM-B3LYP-6-31G(d) [Ligands]/LANL2DZ (Sc)], replacing the Eu atom by a Sc atom to reduce the calculational complexity, can well reproduce the characteristic biphasic CD pattern in this system (Tables S5 and S6).^46^ These modifications enabled us to complete a series of TD-DFT calculations within the limitations of the laboratory-based calculational resources, while preserving the chiral configuration of the three β-diketonate ligands found in the crystal structure (Figures 5b and S6).^59^Figure 5 compares the theoretical CD spectrum of **2** in acetonitrile and its experimental CD spectra produced with isomers **A**–**H**, in which five Cotton bands with a −,+,–,+,– sequence were observed. When we compared the experimental CD spectrum of **2** with the theoretical CD spectrum produced for the most stable isomer **G** (panel a vs panel c of Figure 5), five Cotton bands with a −,+,–,+,– sequence in the experimental CD spectrum (Figure 5a) were successfully reproduced in the theoretical one (Figure 5c). The theoretical CD spectra of the other competing isomers, **A**–**F** and **H,** provide a different CD signal sequence of Cotton bands when compared with the experimental spectra (panel a vs panels d–j of Figure 5). The above emission profile analysis revealed that **2**, with a strongly electron-withdrawing group (R = −CF_3_), exists solely as isomer **G** in acetonitrile (Scheme 2, *vide supra*). Thus, the good agreement between the experimental and theoretical CD patterns (panel a vs c of Figure 5) underlies the validity of the TD-DFT-based structure elucidation (Figure S6). In the present work, such TD-DFT/ECD method should be complementary to the emission spectrum line shape analysis for obtaining information on the configuration of the Eu(III) complexes mainly existing in solution. This study also demonstrated that the electron-withdrawing and -donating effects of the substituents can control the circularly polarized luminescence (CPL) performance of the Eu(III) complexes (Figures S7 and S8 and Table S7).

**Figure 5:**
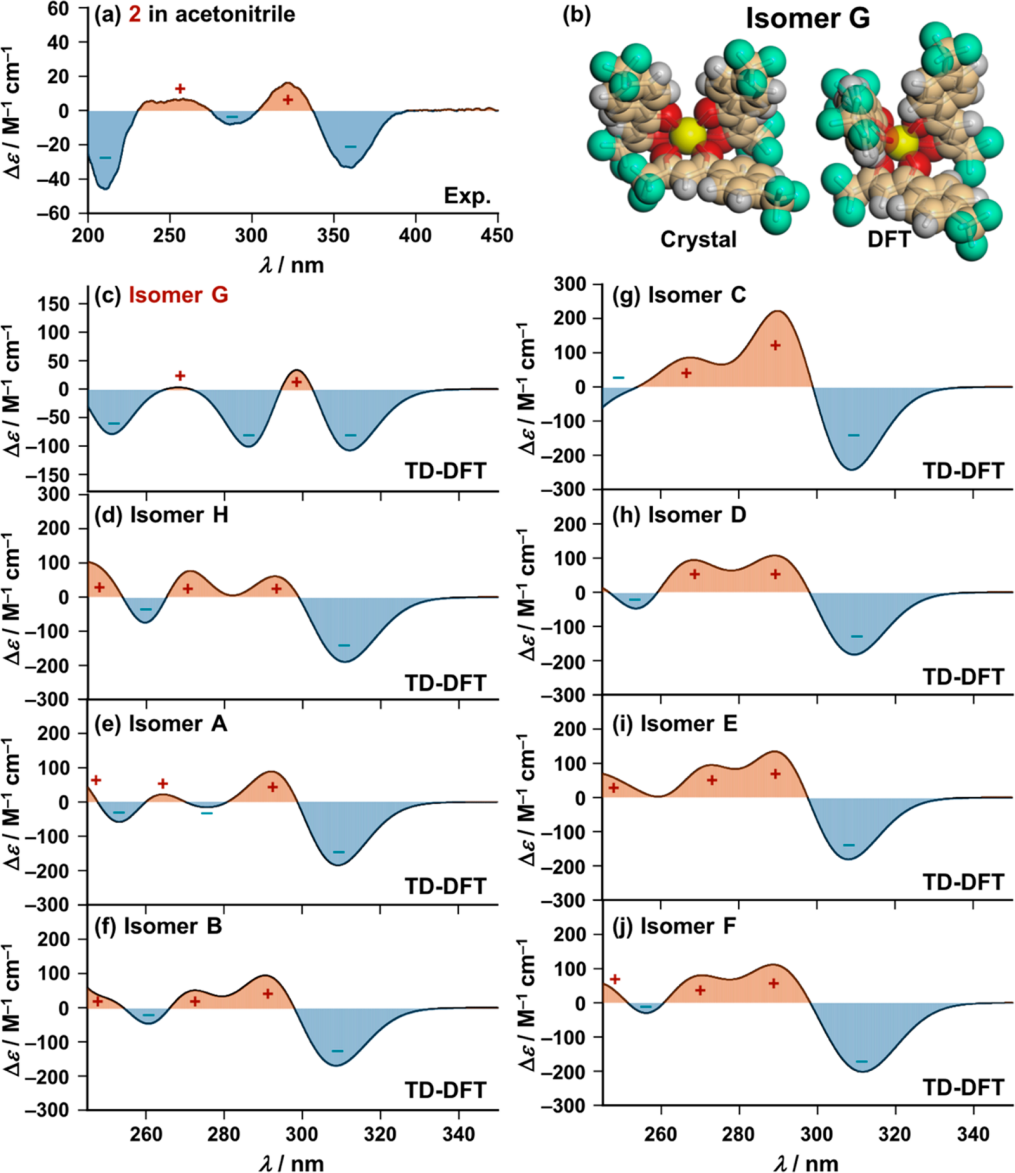
(a) Experimental CD spectrum of **2** (1.0 × 10^–5^ M) in acetonitrile. (b) Arrangement of the three β-diketonate ligands around the Eu(III) metal center found in the crystal structure of **2** and the optimized structure [DFT/CAM-B3LYP-6-31G(d) [C H N O F]/LANL2DZ (Sc)] of **2** (isomer **G**). (c–j) Theoretical CD spectra [TD-DFT/CAM-B3LYP-6-31G(d) [C H N O F]/LANL2DZ (Sc)] of **2** (isomers **A**–**H**), replacing Eu atoms with Sc atoms to reduce the calculation complexity.

In conclusion, we have successfully demonstrated a protocol for determination of the configuration of Eu(III) complexes in solution using the characteristic splitting of the *f*–*f* emission lines caused by crystal-field splitting. The proposed concept can be verified using X-ray crystal structures of nine Eu(III) complexes (**1**–**9**) with appropriate use of DFT-based structure elucidation combined with CD data. The present analytical methodology will pave the way for developing unique lanthanide frameworks formed in solution, which exhibit fascinating photophysical properties.

## Supporting Information Available

Experimental details, crystallographic parameters and refinement details, schematic illustration for extended intercomplex hydrogen bonding, suggested energy difference between the optimized structures, supporting emission spectra, summary of shape measurement analysis, summary of theoretical CD spectrum, electron-withdrawing and -donating effects on CPL, and fundamental photophysical data (PDF)
**1** (CIF)
**2** (CIF)
**3** (CIF)
**4** (CIF)
**5** (CIF)
**6** (CIF)
**7** (CIF)
**8** (CIF)
**9** (CIF)

## Supplementary Material

jz1c01885_si_001.pdf

jz1c01885_si_002.cif

jz1c01885_si_003.cif

jz1c01885_si_004.cif

jz1c01885_si_005.cif

jz1c01885_si_006.cif

jz1c01885_si_007.cif

jz1c01885_si_008.cif

jz1c01885_si_009.cif

jz1c01885_si_010.cif
