# Supplementary material for: Structure Determination of Europium Complexes in Solution Using Crystal-Field Splitting of the Narrow f–f Emission Lines
Source: J Phys Chem Lett. 2021 Jul 19;12(29):6867–74. doi: 10.1021/acs.jpclett.1c01885 (PMC8397343; doi:10.1021/acs.jpclett.1c01885)
Supplement: Supplementary file 1 — jz1c01885_si_001.pdf [file jz1c01885_si_001.pdf]

Supporting Information

Structure Determination of Europium Complexes in Solution Using Crystal-Field Splitting of the Narrow *f*-*f* Emission Lines

Yoshinori Okayasu and Junpei Yuasa*

Graduate School of Chemical Science and Technology, Tokyo University of Science, 1-3 Kagurazaka, Shinjuku-ku, Tokyo 162-8601, Japan.

* To whom correspondence should be addressed.

E-mail: yuasaj@rs.tus.ac.jp

General. Chemicals were purchased from Wako Pure Chemical Indus-tries Ltd. and used as received without further purification. (*R*,*R*)-2,6-bis(4-Phenyl-2-oxazolin-2-yl)pyridine [(*R*)-Ph-Pybox] was obtained from Tokyo Chemical Industry Co., Ltd. (TCI). The emission lifetimes were recorded using a FluoroCube (HORIBA, 3000 U-YSP). The positive ESI mass spectra of the chiral Eu(III) complexes were measured with mass spectrometers (JEOL AccuTOF CS JMS-T100CS for ESI). The emission and UV-vis absorption spectra were measured at room temperature using JASCO FP-6500 and V-660, respectively. CD and CPL spectra were recorded by JASCO J-820 and a tailor-made CPL spectroscopy system,[1] respectively. Emission lifetime, CD and CPL measurements were performed by the presence of 50 eq of (*R*)-Ph-Pybox (5.0×10^{-4} M) in the solutions to avoid dissociation of Ph-Pybox.

Preparation of 1–9. Tris-*β*-diketonate Eu(III) complexes containing electron-withdrawing or -donating substituents were prepared as described in the literature.[2] The (N^N^N*)(O^O')$_3$-type nona-coordinated Eu(III) complexes (**1–9**) were synthesized as follows. Typically, (*R*)-Ph-Pybox (0.17 mmol) and the tris-*β*-diketonate Eu(III) complex (0.17 mmol) containing an electron-donating or withdrawing group were dissolved in methanol (20 mL) in a flask. The reaction mixture was stirred overnight at room temperature. After removing the solvent by evaporation, the obtained white powder was dried under vacuum. The other (N^N^N*)(O^O')$_3$-type Eu(III) complexes were prepared by using the same procedure.

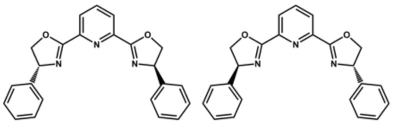

(*R*)-Ph-Pybox (*S*)-Ph-Pybox

1: HRMS (ESI+) *m/z* calcd. for $C_{56}H_{34}F_9N_6O_8EuNa^+$ $[M+Na]^+$ *m/z* = 1265.14044, found 1265.14141.

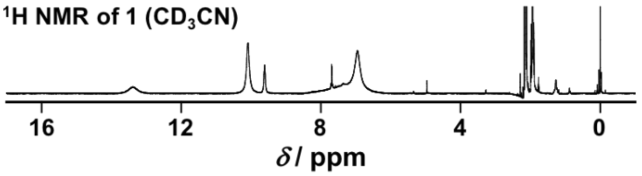

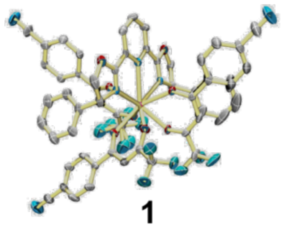

1

2: HRMS (ESI+) *m/z* calcd. for $C_{56}H_{34}F_{18}N_3O_8EuNa^+$ $[M+Na]^+$ *m/z* = 1394.11684, found 1394.11697.

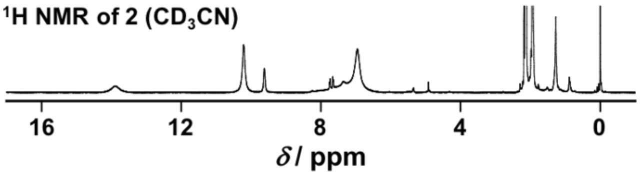

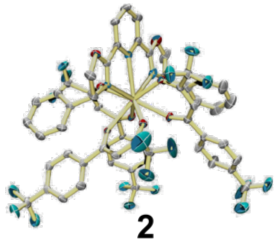

2

3: HRMS (ESI+) *m/z* calcd. for $C_{53}H_{34}F_{12}N_3O_8EuNa^+$ $[M+Na]^+$ *m/z* = 1244.12377, found 1244.12642.

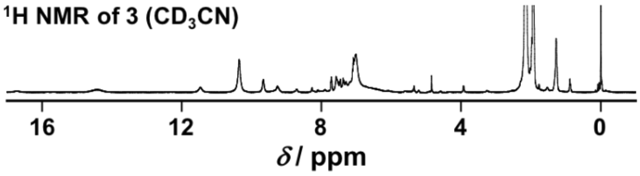

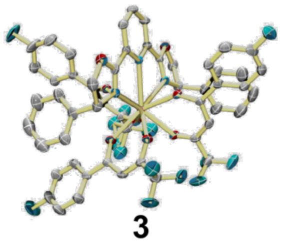

3

4: HRMS (ESI+) *m/z* calcd. for $C_{53}H_{34}Cl_3F_9N_3O_8EuNa^+$ $[M+Na]^+$ *m/z* = 1292.03619, found 1292.03777.

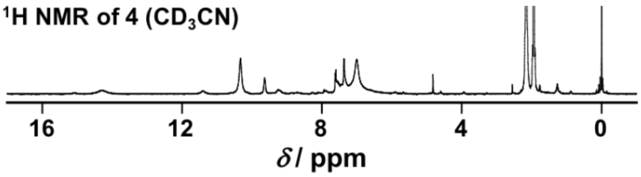

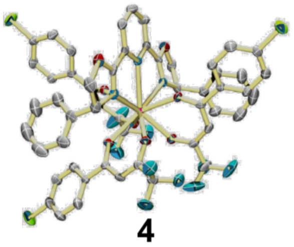

4

5: HRMS (ESI+) *m/z* calcd. for $C_{53}H_{34}Br_3F_9N_3O_8EuNa^+$ $[M+Na]^+$ *m/z* = 1423.88408, found 1423.88623.

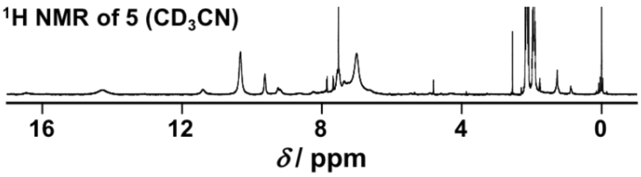

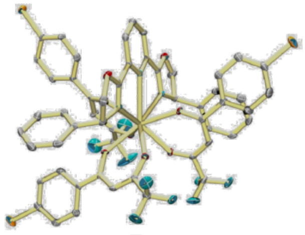

5

6: HRMS(ESI+) *m/z* calcd. for $C_{56}H_{43}EuF_9N_3O_8S_3Na^+$ $[M+Na]^+$ *m/z*= 1326.1159, found 1326.1160.

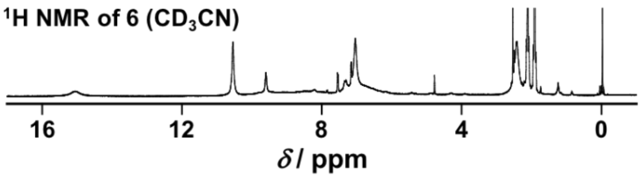

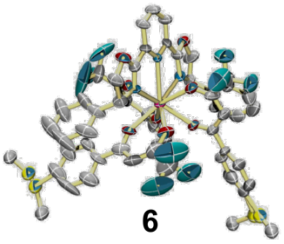

6

7: HRMS(ESI+) *m/z* calcd. for $C_{56}H_{43}EuF_9N_3O_8Na^+$ $[M+Na]^+$ *m/z* = 1230.1997, found 1230.1996.

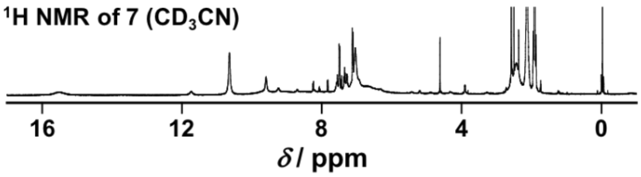

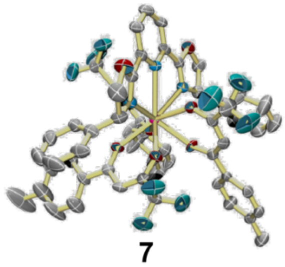

7

8: HRMS(ESI+) *m/z* calcd. for $C_{56}H_{43}EuF_9N_3O_{11}Na^+$ $[M+Na]^+$ *m/z* = 1280.18635, found 1280.18638.

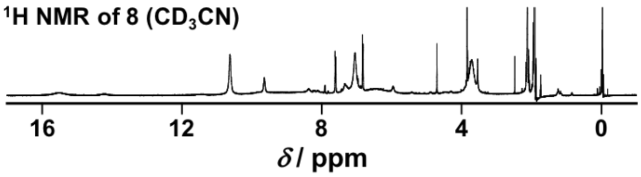

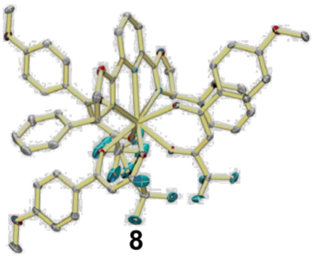

8

9: HRMS(ESI+) *m/z* calcd. for $C_{59}H_{49}EuF_9N_3O_{11}Na^+$ $[M+Na]^+$ *m/z*= 1320.2314, found 1320.2288.

^{1}H NMR of 9 (CD_3CN)

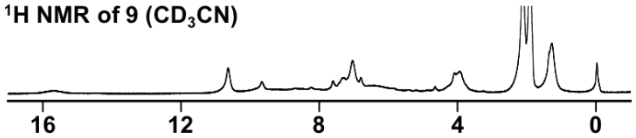

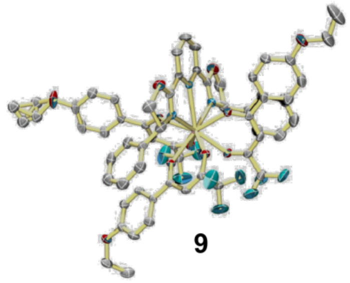

9

Crystal structures and optical properties in solid state.

Crystallography Suitable crystals of **1–9** were obtained by slow evaporation of the acetonitrile solution of **2**, **3**, **4**, **6** and the methanol solution of **1**, **5**, **7**, **8**, **9**. Single crystals of **1–9** were mounted with Dual-Thickness MicroMounts. X-ray diffraction intensity was measured with a Bruker SMART APEXII CCD area detector with graphite monochromated Mo Kα radiation at 100 K or 173 K. All calculations were performed with APEX3 software.

Table S1. Crystallographic data and refinement details for **1**–**5**

	**1**	**2**	**3**	**4**	**5**
**Formula sum**	$C_{56}H_{34}EuF_9$ N_6O_8	$C_{56}H_{34}EuF_{18}$ N_3O_8	$C_{55}H_{37}EuF_{12}$ N_4O_8	$C_{56}H_{34}Cl_3EuF_9N_3O_8$	$C_{53}H_{34}EuF_9$ $N_3O_8Br_3$
**Formula weight**	1241.85	1370.82	1261.84	1306.17	1403.52
**Crystal system**	Triclinic	Monoclinic	Triclinic	Triclinic	Triclinic
**Space group**	*P*1(#1)	$P2_1$(#4)	*P*1(#1)	*P*1(#1)	*P*1(#1)
a **(Å)**	10.2252(15)	12.815(7)	10.079(2)	10.3011(7)	10.198(4)
b **(Å)**	15.936(2)	24.134(14)	11.021(2)	11.0339(7)	10.878(4)
c **(Å)**	16.583(3)	18.953(11)	12.037(3)	12.1085(8)	12.588(5)
a **(deg)**	83.578(2)	90	94.336(2)	93.0010(10)	98.433(6)
b **(deg)**	81.734(2)	107.872(4)	98.721(3)	97.8510(10)	107.288(4)
g **(deg)**	86.630(2)	90	91.7938(18)	92.8650(10)	98.919(4)
V **(Å^3)**	2654.8(7)	5579(6)	1316.7(5)	1359.23(16)	1289.4(9)
T **(K)**	173	173	173	173	100
Z	2	4	1	1	1
F_{000}	1240.0	2720.0	630.0	650.0	686.0
r_{calcd} **(g cm^{-3})**	1.553	1.632	1.292	1.596	1.808
R_1 **[I > 2s(I)]**	0.0322	0.0354	0.0212	0.0229	0.0216
**wR_2 [I > 2s(I)]**	0.0733	0.0752	0.0491	0.0514	0.0461

Table S2. Crystallographic data and refinement details for **6**–**9**

	**6**	**7**	**8**	**9**
**Formula sum**	$C_{56}H_{43}EuF_9N_3O_8S_3$	$C_{56}H_{43}EuF_9N_3O_8$	$C_{56}H_{43}Eu\ F_9N_3O_{11}$	$C_{59}H_{49}EuF_9N_3O_{11}$
**Formula weight**	1305.07	1208.89	1256.89	1298.97
**Crystal system**	tetragonal	Monoclinic	Triclinic	Orthorhombic
**Space group**	$P4_12_12$(#92)	$P2_1$(#4)	$P1$(#1)	$P2_12_12_1$(#19)
a **(Å)**	17.2083(15)	13.0611(10)	10.396(7)	14.844(8)
b **(Å)**	17.2083(15)	38.480(3)	11.164(7)	18.882(10)
c **(Å)**	37.945(3)	16.0189(13)	12.730(8)	20.014(10)
a **(deg)**	90	90	100.881(10)	90
b **(deg)**	90	90.7030(10)	109.334(9)	90
g **(deg)**	90	90	97.095(12)	90
V **(Å³)**	11236(2)	8050.3(11)	1241.2(15)	5610(5)
T **(K)**	100	173	100	173
Z	8	6	1	4
F_{000}	5176.0	3648.0	632.0	2624.0
r_{calcd} **(g cm⁻³)**	1.532	1.496	1.556	1.538
R_1 **[I > 2s(I)]**	0.0573	0.0373	0.0232	0.0224
wR_2 **[I > 2s(I)]**	0.1458	0.0730	0.0504	0.0545

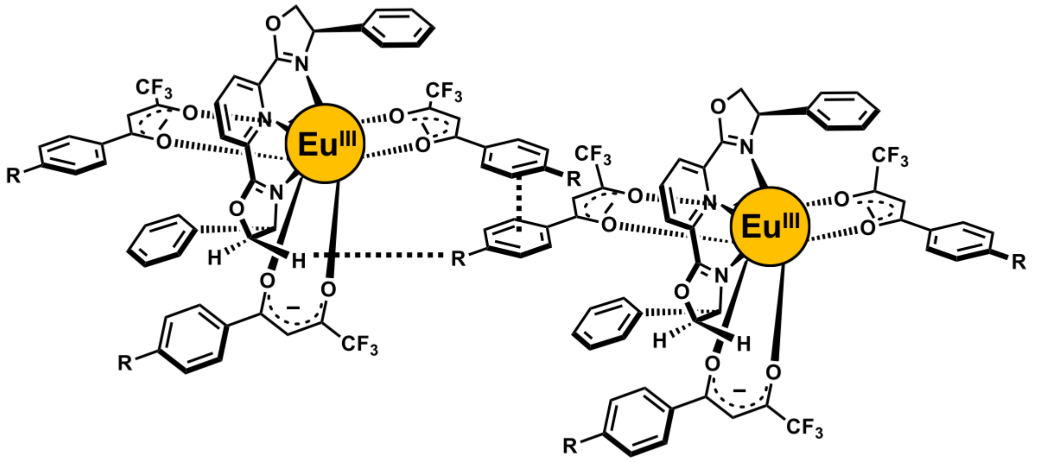

Figure S1. Schematic illustration for extended inter-complex hydrogen bonding found in the X-ray crystal structure of isomer **H**.

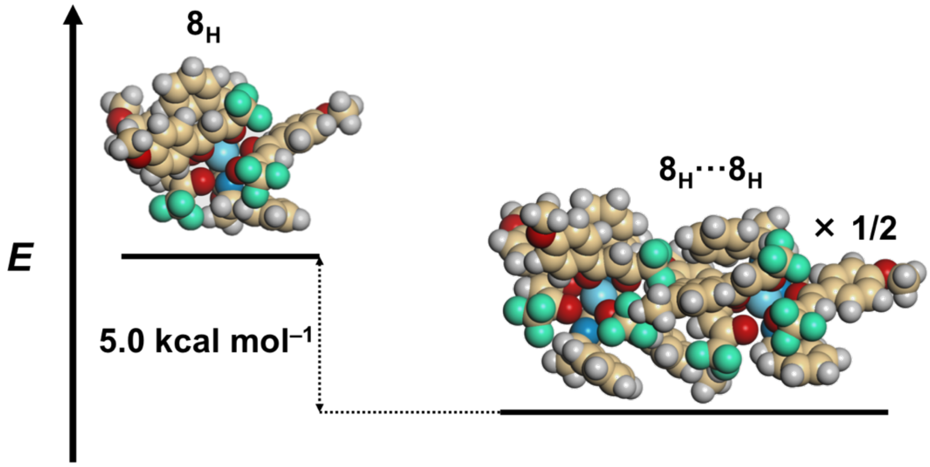

Figure S2. Suggested energy difference between the optimized structures [DFT/CAM-B3LYP/def2SVP (ligands)/def2TZVPP (La)] of $\mathbf{8_H}$ and $\mathbf{8_H}\cdots\mathbf{8_H}$.

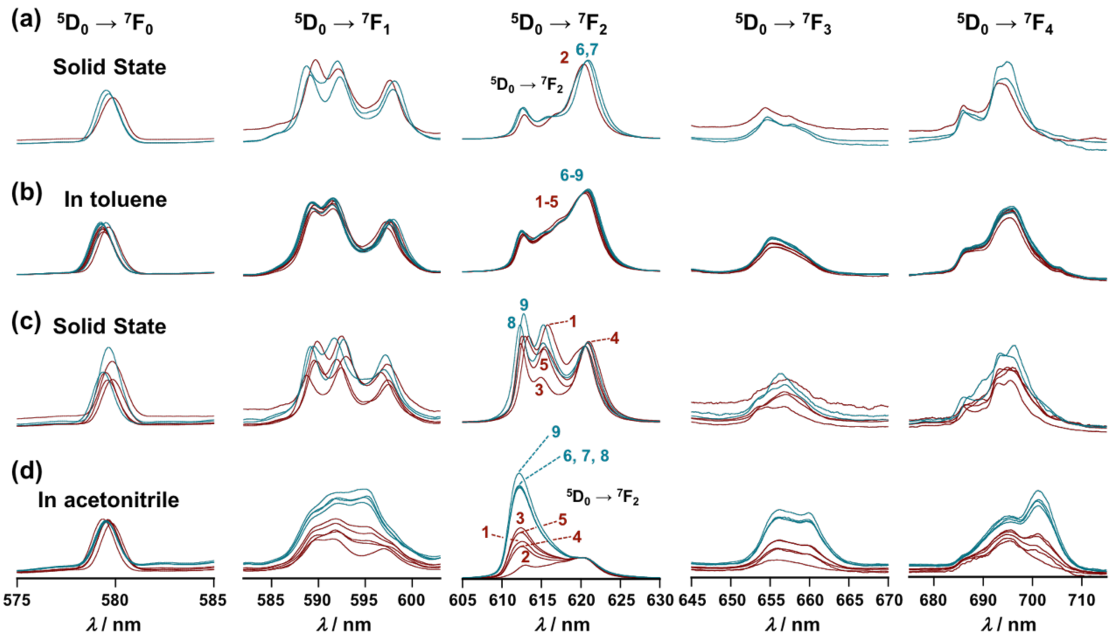

Figure S3. (a,c) Solid-state emission spectra (KBr) of (a) **2**, **6**, and **7**, (c) **1**, **3**, **4**, **5**, **8**, and **9**. (b,d) Emission spectra of **1–9** (concentrations: 1.0×10^{-5} M) (b) in toluene and (d) in acetonitrile.

Table S3. Summary of shape measures analysis for the crystal structures of **1**–**5** (basic shape of coordination sphere: capped square antiprism)

Geometry	**1**	**2**	**3**	**4**	**5**
Enneagon (D_{9h})	26.042	28.288	31.477	31.046	29.148
Octagonal pyramid (C_{8v})	21.990	21.264	22.245	22.724	20.000
Heptagonal bipyramid (D_{7h})	22.923	24.207	18.866	19.072	18.095
Johnson triangular cupola J3 (C_{3v})	15.104	21.139	14.942	14.644	15.916
Capped cube J8 (C_{4v})	9.633	16.849	8.649	8.644	11.360
Spherical-relaxed capped cube (C_{4v})	12.233	16.896	8.968	8.780	11.200
Capped square antiprism J10 (C_{4v})	6.347	10.212	2.080	2.258	4.980
Spherical capped square antiprism (C_{4v})	7.908	8.686	2.301	2.343	4.940
Tricapped trigonal prism J51 (D_{3h})	5.634	10.293	2.575	2.361	4.420
Spherical tricapped trigonal prism (D_{3h})	8.558	9.238	2.216	2.092	5.288
Tridiminished icosahedron J63 (C_{3v})	15.516	19.070	14.497	14.043	13.869

Table S4. Summary of shape measures analysis for the crystal structures of **6**–**9** (basic shape of coordination sphere: capped square antiprism)

Geometry	**6**	**7**	**8**	**9**
Enneagon (D_{9h})	19.419	21.637	28.894	34.460
Octagonal pyramid (C_{8v})	10.403	13.475	19.151	19.902
Heptagonal bipyramid (D_{7h})	21.435	21.009	17.366	18.281
Johnson triangular cupola J3 (C_{3v})	16.538	19.012	16.614	17.001
Capped cube J8 (C_{4v})	17.203	19.752	12.364	11.906
Spherical-relaxed capped cube (C_{4v})	16.969	19.968	11.475	9.922
Capped square antiprism J10 (C_{4v})	11.323	14.904	5.812	4.075
Spherical capped square antiprism (C_{4v})	8.998	13.453	5.280	2.546
Tricapped trigonal prism J51 (D_{3h})	11.199	13.963	5.123	5.644
Spherical tricapped trigonal prism (D_{3h})	8.565	13.440	6.044	1.133
Tridiminished icosahedron J63 (C_{3v})	17.335	19.672	13.748	11.634

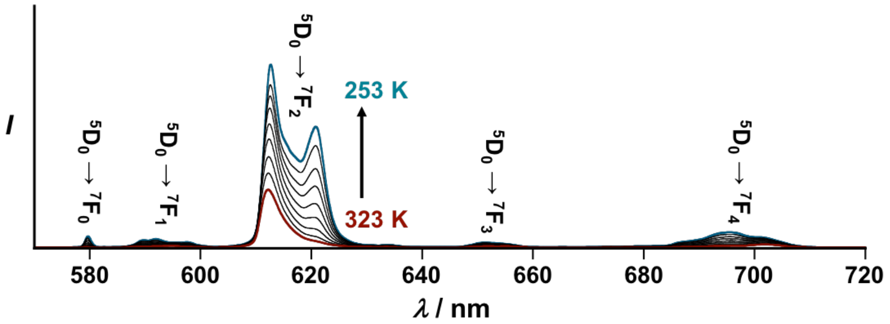

Figure S4. Emission spectra of **8** (1.0×10^{-5} M) in acetonitrile at 253–323 K (blue line–red line).

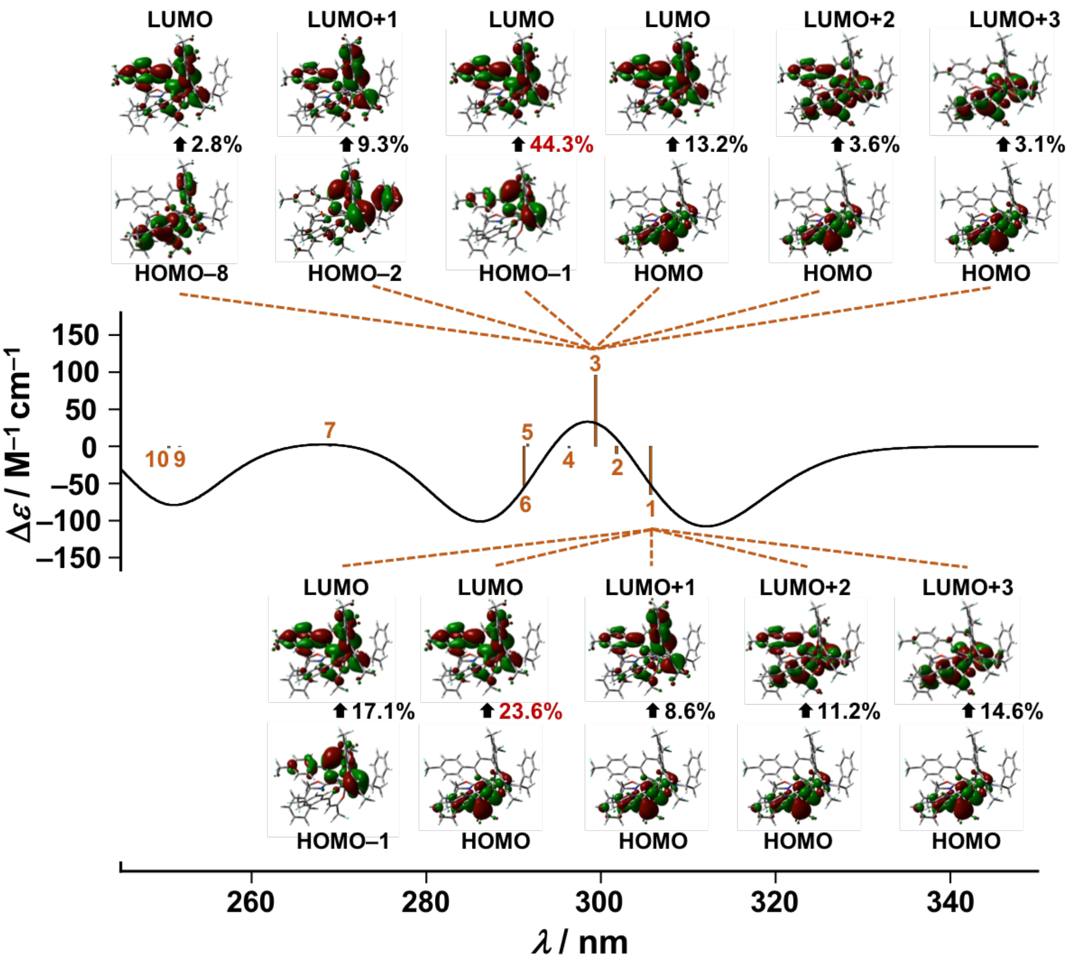

Figure S5. Theoretical CD spectrum [time dependent-DFT/CAM-B3LYP-6-31G(d) [C H N O F]/LANL2DZ (Sc)] of **2** (isomers **G**), where Eu atoms are replaced by Sc atoms to reduce the calculation complexity. Negative and positive Cotton bands arising from excited states 1 and 3 are attributed by electron transitions between the three chromophore ligands, indicating that biphasic CD spectrum is result of excitonic coupling between the chromophore ligands.

Table S5. Summary of theoretical CD spectra [TD-DFT/CAM-B3LYP-6-31G(d) [C H N O F]/LANL2DZ (Sc)] of **2** (isomers **A**–**H**)

Isomer	Arrangement of the *β*-diketonate ligands	CD sign Sequence
Isomer **A**	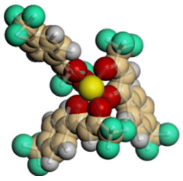	–,+,–,+,–,+
Isomer **B**	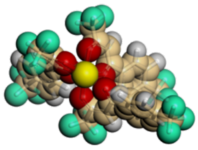	–,+,+,–,+
Isomer **C**	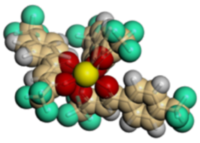	–,+,+,–
Isomer **D**	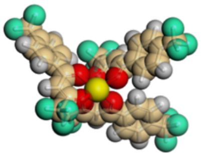	–,+,+,–
Isomer **E**	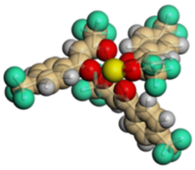	–,+,+,+
Isomer **F**	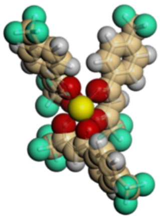	–,+,+,–,+
Isomer **G**	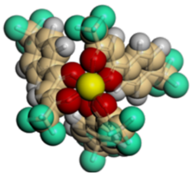	–,+,–,+,–
Isomer **H**	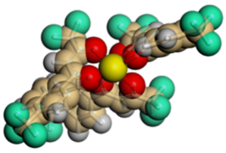	–,+,+,–,+

Table S6. Summary of theoretical CD spectra [TD-DFT/CAM-B3LYP-6-31G(d) [C H N O F]/LANL2DZ (Sc)] of **8** (isomers **A**–**H**)

Isomer	Arrangement of the β-diketonate ligands	CD sign Sequence
Isomer **A**	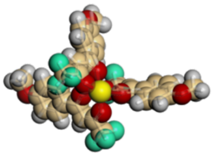	–,+,+,–
Isomer **B**	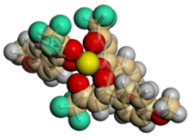	–,+,+,+,–
Isomer **C**	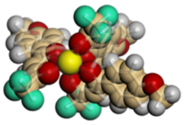	–,+,+,–,+
Isomer **D**	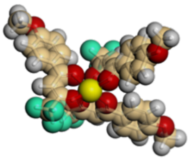	–,+,+,–,+,–
Isomer **E**	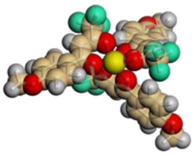	–,+,–,–
Isomer **F**	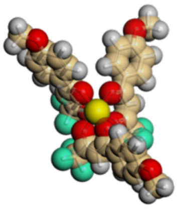	–,+,–,–
Isomer **G**	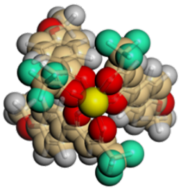	–,+,–,+,–
Isomer **H**	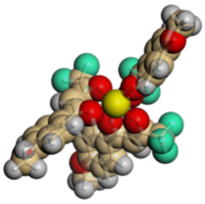	–,+,+,+,–

TD-DFT-based structure elucidation on 8 (S6): The CD spectrum of **8** in acetonitrile also gave rise to five Cotton bands with a –,+,–,+,– sequence (Fig. S6a), reproducing well the theoretical CD spectrum of **8** with the configuration of isomer **G** (Fig. S6a vs S6c). In the present case, the above emission profile analysis suggests that **8**, with an electron-donating group (R = -OMe), involves not only the most stable isomer **G** but also the competing isomers in acetonitrile (Scheme 2d, vide supra). The observed agreement between the experimental and theoretical CD patterns (Fig. S6a vs S6c) is probably due to a smaller contribution of the competing isomers on the observed CD spectrum, or due to the similarity of the CD pattern of the competing isomers, such as isomer **A**, to that of isomer **G** (Figs. S6c and S6e). Hence, further comprehensive analysis should be required for the structural determination of the species in solution in such cases.

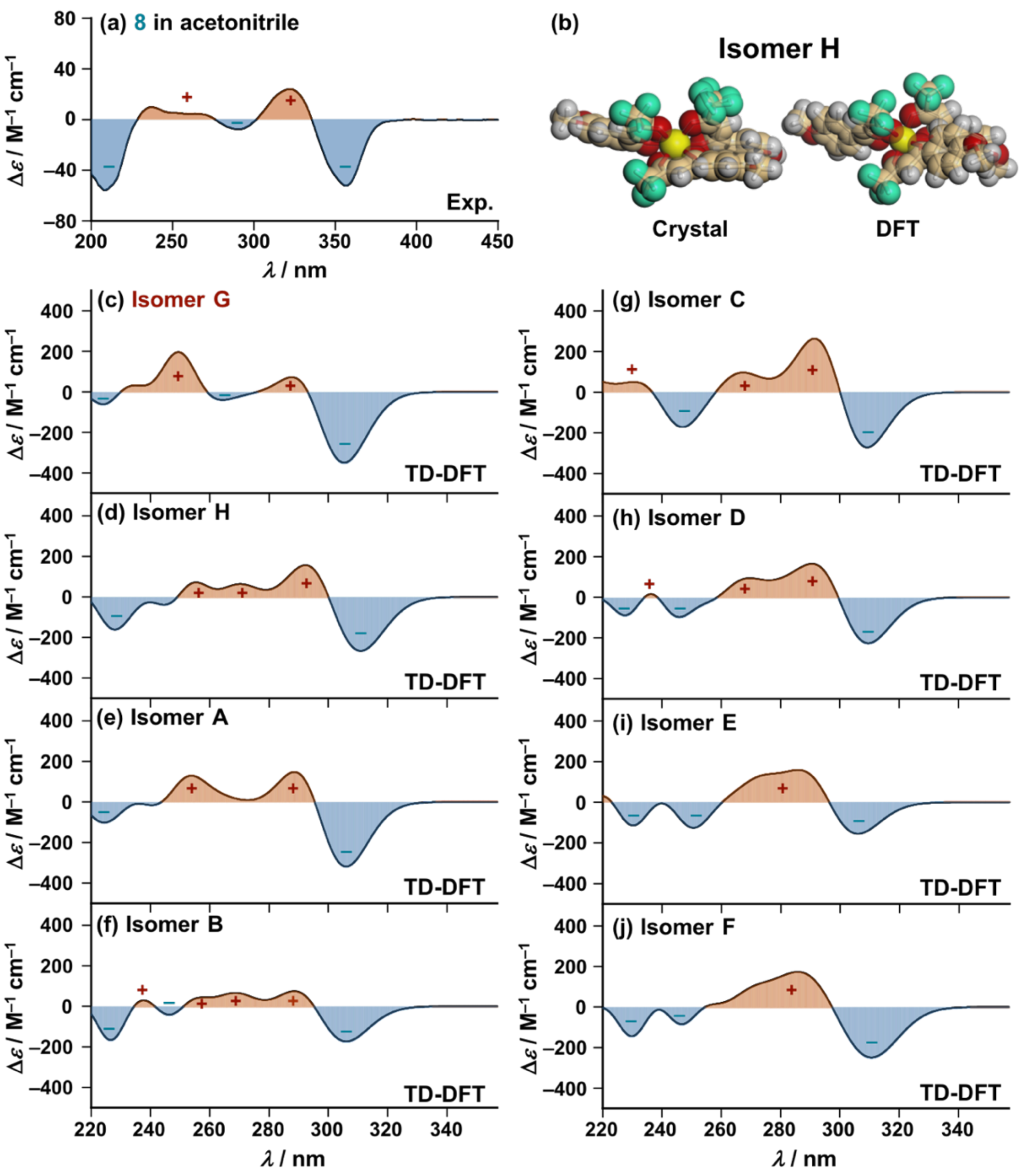

Figure S6. (a) Experimental CD spectrum of **8** (1.0×10^{-5} M) in acetonitrile. (b) Arrangement of the three *β*-diketonate ligands around the Eu(III) metal center found in the crystal structure of **8** and the optimized structure [DFT/CAM-B3LYP-6-31G(d) [C H N O F]/LANL2DZ (Sc)] of **8** (isomer **H**). (c-j) Theoretical CD spectra [TD-DFT/CAM-B3LYP-6-31G(d) [C H N O F]/LANL2DZ (Sc)] of **8** (isomers **A**–**H**), replacing Eu atoms with Sc atoms to reduce the calculational complexity.

Electron-Withdrawing and -Donating Effects on Circularly Polarized Luminescence (CPL) (S7 and S8): We also investigated whether the electron-withdrawing and -donating ability of the substituents attached to the *β*-diketonate ligands affects the circularly polarized luminescence (CPL) performance of the Eu(III) core inside the complex. In the nonpolar solvent toluene and the polar solvent acetonitrile, all complexes **1**–**9** exhibited detectable CPL at the $^5D_0 \rightarrow {}^7F_1$ transition band using our tailor-made CPL measuring system (Fig. S7).[1,3] The luminescence dissymmetry factor ($g_{lum} = 2(I_L - I_R)/(I_L + I_R)$) was plotted against the Hammett constants of the electron-withdrawing and -donating substituents on the ligands (I_L and I_R denote the intensity of the left and right CPL, respectively). First, the g_{lum} values are almost constant, around g_{lum} ~0.16 in the non-polar solvent toluene, irrespective of the electron-withdrawing or -donating ability of the substituents (Fig. 6a). The above structure analysis revealed that all complexes **1**–**9** in toluene exist solely as isomer **G** (vide supra, Scheme 2a). Hence, the identical g_{lum} of about 0.16 obtained for a wide range of Eu(III) complexes (**1**–**9**) in the non-polar solvent (Fig. S8a) suggests that the electron-withdrawing and -donating effects of the substituents have essentially no impact on the CPL performance of the Eu(III) core inside the complex. In the polar solvent acetonitrile, g_{lum} increased with an increase in the electron-withdrawing ability of the substituents to approach the identical g_{lum} value (~0.16) obtained in toluene (Fig. S8b). The above structure analysis suggests that the competing isomers co-exist with isomer **G** in acetonitrile; the relative ratio of the competing isomers to isomer **G** decreases with increasing electron-withdrawing ability of the outer substituents (vide supra, Scheme 2c and d). Therefore, the observed change in g_{lum} (Fig. S8b) can be explained rationally if the competing isomers exhibit smaller g_{lum} values than that of isomer **G** (Scheme 2c and d). Thus, the substituents attached to the chromophoric ligands are capable of exerting external control in the CPL performance of the Eu(III) complexes in solution. In most cases, the g_{lum} values at the $^5D_0 \rightarrow {}^7F_2$ transition band are much smaller (~1/10) than those at the $^5D_0 \rightarrow {}^7F_1$ transition band. Although our CPL measuring system is not accessible for accurate determination of the smaller g_{lum} values, there is a good relationship between the g_{lum} values at the $^5D_0 \rightarrow {}^7F_1$ and $^5D_0 \rightarrow {}^7F_2$ transition bands, where the Eu(III) complex with larger g_{lum} value at the $^5D_0 \rightarrow {}^7F_1$ transition has a tendency to give larger g_{lum} value at $^5D_0 \rightarrow {}^7F_2$ transition band (with opposite sign). Thus, similar results as the g_{lum} values at the $^5D_0 \rightarrow {}^7F_1$ transition (Figure S8) should be obtained with those at $^5D_0 \rightarrow {}^7F_2$ transition.

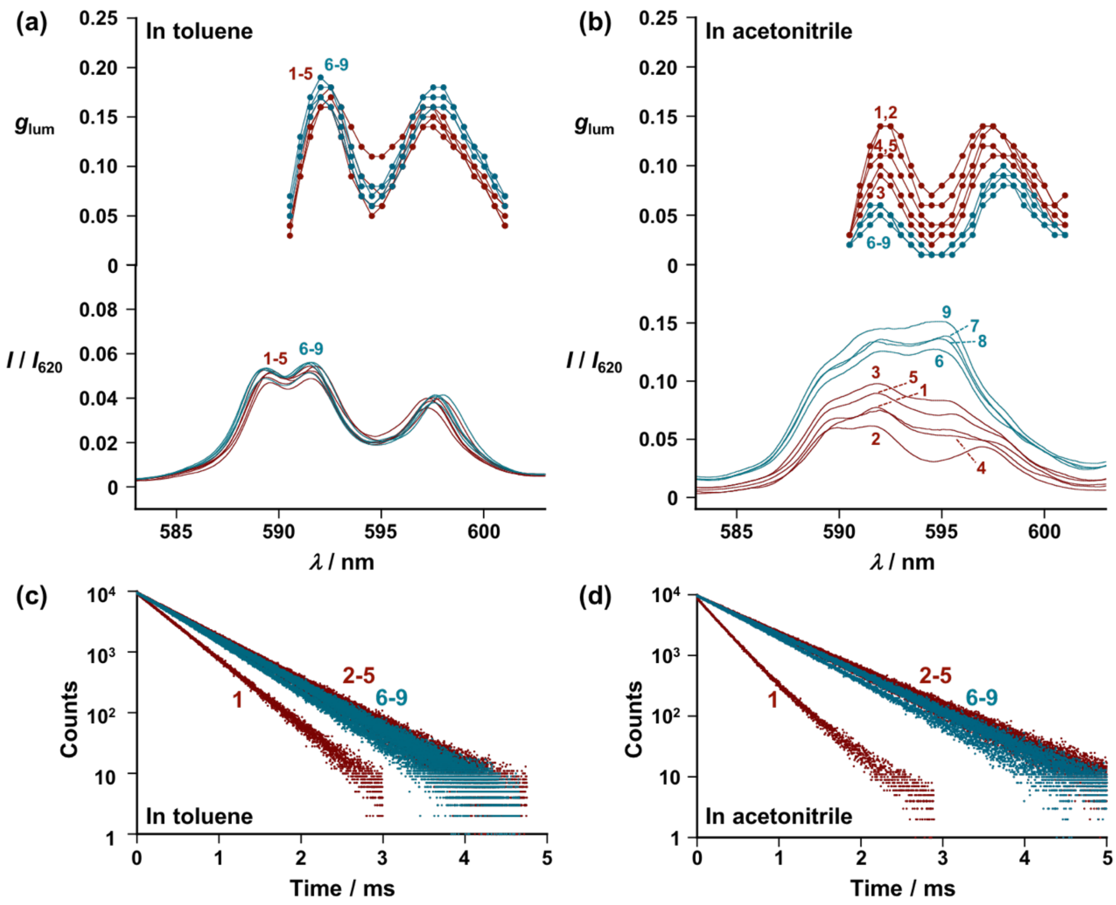

Figure S7. CPL (top) and emission (bottom) spectra of **1–9** (concentration: 1.0×10^{-5} M) in (a) toluene and (b) acetonitrile. Emission decay profiles of **1–9** (concentration: 1.0×10^{-5} M) at λ = 612 nm in acetonitrile at 298 K. Excitation wavelength: λ_{ex} = 371 nm.

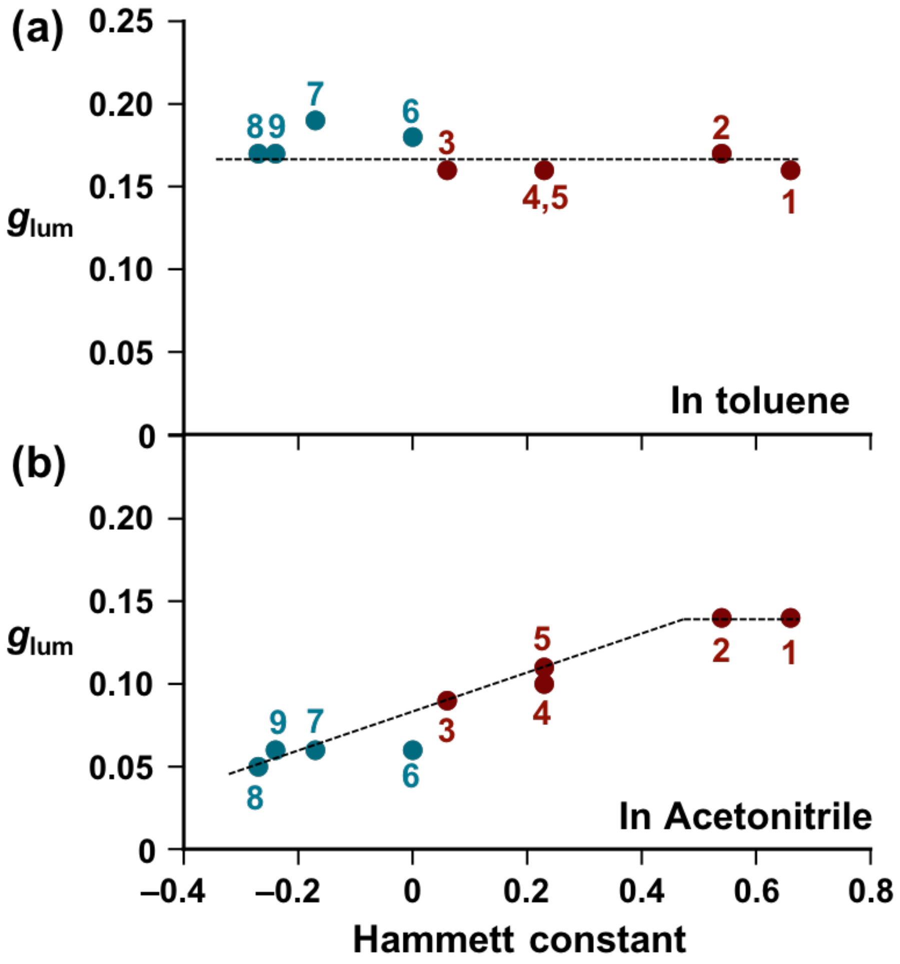

Figure S8. Plots of g_{lum} of **1**–**9** (concentrations: 1.0×10^{-5} M) at 592 nm in (a) toluene and (b) acetonitrile versus the Hammett constant of the electron-withdrawing and -donating substituents attached to the β-diketonate ligands.

Table S7. Fundamental photophysical data of **1–9**

Complex	g_{lum}[a]	g_{lum}[b]	τ_{obs}[a] (ms)	τ_{obs}[b] (ms)	ϕ_{em}[b,c,d] (%)
**1**	0.16	0.14	0.40	0.27	9
**2**	0.17	0.14	0.56	0.60	24
**3**	0.16	0.09	0.62	0.73	27
**4**	0.16	0.11	0.59	0.70	30
**5**	0.16	0.10	0.58	0.65	25
**6**	0.18	0.06	0.53	0.61	22
**7**	0.19	0.06	0.59	0.68	15
**8**	0.17	0.06	0.58	0.67	9
**9**	0.17	0.05	0.57	0.66	11

[a] Determined in toluene. [b] Determined in acetonitrile. [c] The value upon excitation of the chromophore ligands. [d] Determined by integration sphere.

References

(1) Okayasu, Y.; Yuasa, J. *Mol. Syst. Des. Eng.* **2018**, *3*, 66.

(2) Reid, J. C.; Calvin, M. *J. Am. Chem. Soc.* **1950**, *72*, 2948.

(3) Okayasu Y.; Yuasa, J. *Kidorui* **2020**, *76*, 62.
